# A Novel Food Record App for Dietary Assessments Among Older Adults With Type 2 Diabetes: Development and Usability Study

**DOI:** 10.2196/14760

**Published:** 2021-02-17

**Authors:** Hyunggu Jung, George Demiris, Peter Tarczy-Hornoch, Mark Zachry

**Affiliations:** 1 Department of Computer Science and Engineering University of Seoul Seoul Republic of Korea; 2 Department of Artificial Intelligence University of Seoul Seoul Republic of Korea; 3 Department of Biobehavioral and Health Sciences University of Pennsylvania Philadelphia, PA United States; 4 Department of Biomedical Informatics and Medical Education University of Washington Seattle, WA United States; 5 Division of Neonatology, Department of Pediatrics University of Washington Seattle, WA United States; 6 Department of Computer Science and Engineering University of Washington Seattle, WA United States; 7 Department of Human Centered Design and Engineering University of Washington Seattle, WA United States

**Keywords:** mobile health, older adults, diabetes, dietary assessment, smartphone app, usability test

## Abstract

**Background:**

More than 1 in 4 people in the United States aged 65 years and older have type 2 diabetes. For diabetes care, medical nutrition therapy is recommended as a clinically effective intervention. Previous researchers have developed and validated dietary assessment methods using images of food items to improve the accuracy of self-reporting over traditional methods. Nevertheless, little is known about the usability of image-assisted dietary assessment methods for older adults with diabetes.

**Objective:**

The aims of this study were (1) to create a food record app for dietary assessments (FRADA) that would support image-assisted dietary assessments, and (2) to evaluate the usability of FRADA for older adults with diabetes.

**Methods:**

For the development of FRADA, we identified design principles that address the needs of older adults and implemented three fundamental tasks required for image-assisted dietary assessments: capturing, viewing, and transmitting images of food based on the design principles. For the usability assessment of FRADA, older adults aged 65 to 80 years (11 females and 3 males) were assigned to interact with FRADA in a lab-based setting. Participants’ opinions of FRADA and its usability were determined by a follow-up survey and interview. As an evaluation indicator of usability, the responses to the survey, including an after-scenario questionnaire, were analyzed. Qualitative data from the interviews confirmed the responses to the survey.

**Results:**

We developed a smartphone app that enables older adults with diabetes to capture, view, and transmit images of food items they consumed. The findings of this study showed that FRADA and its instructions for capturing, viewing, and transmitting images of food items were usable for older adults with diabetes. The survey showed that participants found FRADA easy to use and would consider using FRADA daily. The analysis of the qualitative data from interviews revealed multiple categories, such as the usability of FRADA, potential benefits of using FRADA, potential features to be added to FRADA, and concerns of older adults with diabetes regarding interactions with FRADA.

**Conclusions:**

This study demonstrates in a lab-based setting not only the usability of FRADA by older adults with diabetes but also potential opportunities using FRADA in real-world settings. The findings suggest implications for creating a smartphone app for an image-assisted dietary assessment. Future work still remains to evaluate the feasibility and validity of FRADA with multiple stakeholders, including older adults with diabetes and dietitians.

## Introduction

### Older Adults with Type 2 Diabetes

Approximately 1 in 10 people in the United States has type 2 diabetes. More than 1 in 4 Americans aged 65 years and older have type 2 diabetes [1]. As the population of individuals aged 65 or over is anticipated to reach approximately 74 million by 2030 [2], it is expected that the population of older adults with diabetes will increase accordingly. Diabetes has a tremendous impact on the health of the US population. In 2013, it was reported that diabetes was the seventh leading cause of death [3]. Also, it is known that diabetes increases the risk of heart attack and stroke [1], as well as the risk of cancer, especially colorectal cancer [4-6]. Diabetes has a disproportionate impact on the health of older adults. Diabetes is known as the leading cause of blindness and kidney failure in older adults. Older adults with diabetes are two times more likely to develop dementia than older adults without diabetes [7]. Furthermore, 1 in 5 people aged 65 years and older has vision problems [7], and 1 in 3 adults with diabetes may have chronic kidney disease. Moreover, it is known that people over 75 years of age with diabetes are two times more likely to visit the emergency room for low blood glucose than younger patients with diabetes [7]. Diabetes also takes a financial toll. The estimated total costs of diagnosed diabetes in the United States rose to $327 billion in 2017 from $245 billion in 2012, which was a 41% increase in a 5-year period [8,9]. The total costs in 2017 included $237 billion for direct medical costs and $90 billion for indirect costs, such as inability to work as a result of disease-related disability, reduced productivity for those not in the labor force, and lost productive capacity due to early mortality [8,9]. The estimated costs imply that diabetes produces explicit and inexplicit burdens to both individual patients and society as a whole.

### Medical Nutrition Therapy (MNT)

To improve diabetes care, the American Diabetes Association suggests a multipronged strategy to support the patients’ behavior change efforts including healthy lifestyle changes (eg, physical activity, healthy eating, tobacco cessation, weight management, and effective coping), disease self-management, and prevention of diabetes complications [10]. MNT is recommended for individuals with diabetes as part of their overall treatment plan [3] to achieve the goals of nutritional therapy [11]. In particular, MNT is recommended as a clinically effective model to take care of individuals with diabetes [12-15], including older adults [16]. In order to meet treatment goals, individuals with diabetes are required to receive personalized MNT from registered dietitians and nutrition professionals [3].

### Traditional Dietary Assessment Methods

For dietary advice, it is essential for dietitians to assess the nutritional status of patients with a variety of dietary data, such as meal patterns, food choices, and overall dietary balance. To collect such dietary data from each patient, dietitians use methods such as food records and 24-hour dietary recall (24HR). The food records method is an approach in which the patient is asked to write down all food items and the amounts consumed over one or more days. The objective of this method is to obtain a detailed description of food intake, including types and amounts of foods and beverages they have consumed. Since this method allows respondents to record their food intake right after they have consumed it, they do not have to rely on recalled memories of their meals. On the other hand, the 24HR method is an approach to get retrospective information about food consumption patterns through interviews with the patient. While the food records method needs some level of literacy to produce the records, the 24HR method does not require knowing how to describe food items to dietitians because a dietitian speaks with the respondent directly during the interview.

However, it is difficult to obtain accurate nutritional information using these methods because they are based on self-reported data. For instance, respondents would need to have strong motivation and literacy to keep recording their food intake using the food records method. Individuals tend not to maintain regular performance on such tasks over long periods [17]. Instead, they might prefer recording the food items for three meals at the same time based on memory instead of documenting the information every single time. In addition, the 24HR method requires individuals to recall the food items they consumed and the specific amounts they consumed [18]. Individuals might forget to mention all of the food items. Additionally, they might have trouble identifying the contents of the food items and estimating portion sizes. It might be particularly difficult to collect reliable data from older adults using traditional methods for dietary assessment because they have special considerations (eg, dietary restrictions) and diminished functional statuses. For example, the 24HR method might be inappropriate because memories among older adults are more likely to be impaired than those of younger adults [19,20]. In one study, older adults did not report energy intake adequately during the 24HR assessment [21].

### Dietary Assessment Methods Using Food Images

To overcome the limitations of self-reporting by traditional methods for a dietary assessment, researchers have developed and validated dietary assessment methods using images of food items to improve the accuracy of self-reporting over traditional methods. Prior studies demonstrated the benefits of using images of food items. For example, they revealed that the use of images of food items led to identifying unreported foods and misreporting errors [22-24]. The dietary information from the images enabled researchers to identify additional energy intake of the given food items [22-24].

In other studies, researchers evaluated image-assisted 24HR methods using a variety of devices (Table 1). Prior studies not only showed how image-assisted dietary assessment methods reduced errors in self-reported data but also validated the use of an image-assisted dietary assessment method with general populations. Four studies recruited healthy adults [22,25-27] and the maximum mean age of the participants across the studies was 35 years [22,25,27-29]. One study included adults with intellectual and developmental disabilities [30], but no studies recruited participants with any chronic diseases nor had participants aged 65 years or older to evaluate the image-assisted dietary assessment methods. The sample sizes of prior studies were small: four studies had between 20 and 54 participants [26-29] and two studies had fewer than 20 participants [22,25]. In regard to the approach used to capture images, three studies used passive image capture with wearable cameras [22,25,28], while three studies used active image capture with a handheld digital camera [27], iPad 2 (Apple Inc) [29], or smartphone [26].

**Table 1:** Characteristics of image-assisted 24-hour dietary recall methods.

Study	Participants	Age of participants (years), mean (SD)	Device for image collection	Capture	View	Transmit
[25]	14 healthy adults	35 (12)	Mobile phones with camera	✓	✓	✓
[27]	43 healthy adult women	35 (9)	Digital camera	✓	✓	Not reported
[22]	10 healthy adults	33 (11)	SenseCam	✓	✓	✓
[29]	23 adults with intellectual and developmental disabilities	26.4 (9.7)	iPad 2 (Apple Inc)	✓	✓	Not reported
[26]	54 healthy adults	Range: 19-28	Smartphone	✓	✓	✓
[28]	40 adults (20 females, 20 males)	Females: 28 (7); males: 35 (17)	SenseCam	✓	✓	✓

We found two gaps after reviewing those studies. First, no studies have evaluated the usability of image-assisted dietary assessment methods with older adults with diabetes. Second, little is still known about the usability of the image-assisted dietary assessment methods using smartphone apps, although a smartphone is the type of device that could perform the following three fundamental tasks required for image-assisted dietary assessments: (1) capturing images of food and beverages, (2) viewing images of food and beverages, and (3) transmitting images of food and beverages. Table 1 shows the characteristics of image-assisted 24HR methods.

To address those gaps, we aimed to answer multiple research questions through surveys and interviews (see Textbox 1). The specific aims of our study were (1) to develop a smartphone app for image-assisted dietary assessments, and (2) to evaluate the usability of the app by older adults with diabetes. Specifically, no data on the accuracy of identifying foods and estimating portion sizes were reported in this study.

**Research questions we aimed to answer through surveys and interviews.**

- RQ1: How likely is it that older adults with diabetes will be satisfied with the ease of completing each task (ie, capturing, viewing, and transmitting images)?
- RQ2: How likely is it that older adults with diabetes will be satisfied with the amount of time it took to complete each task by using a food record app for dietary assessments (FRADA)?
- RQ3: What is the usability of FRADA with older adults with diabetes?
- RQ4: Would older adults with diabetes use a tablet as an alternative device?
- RQ5: Would it be easy for older adults with diabetes to follow the instructions, such as including all the food items in one single photograph and holding the phone at a 45-degree angle when taking pictures of food items?
- RQ6: Would older adults with diabetes want to use FRADA in the future?
- RQ7: What are the potential benefits for older adults with diabetes of using FRADA?
- RQ8: What are the potential features to be added to FRADA?
- RQ9: What are the concerns of older adults with diabetes when interacting with FRADA?
- RQ10: What are the potential target populations of FRADA?

## Methods

### Development of a Food Record App for Dietary Assessments

The first goal of this study was to create a food record app for dietary assessments (FRADA) to enable older adults with diabetes to collect images of their meals and snacks. Since collected images will be reviewed by dietitians in real-life scenarios, we developed two subapps of FRADA for older adults with diabetes: a client app and a server app to view collected images. Also, we developed an app that enables dietitians to view images of food items transmitted by smartphones of older adults with diabetes during 24HR interviews.

### Development of Client and Server Apps for Older Adults With Diabetes

**Figure 1:**
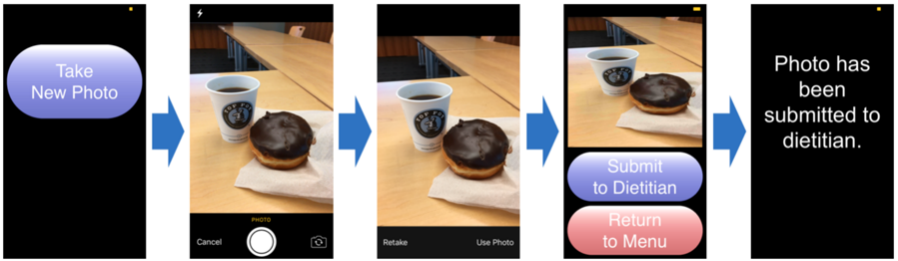
Screenshots of the user interface in the smartphone app.

The role of the client app is for older adults with diabetes to transmit collected images of food items to a server from their smartphone; therefore, this app was embedded in their smartphones. On the other hand, we implemented another subapp on the server, which receives transmitted images of food items, stores them, and manages any requests from the client side in the server. We used HTML and JavaScript languages to develop the client app using Apache Cordova [31] and a PHP language [32] to implement the app on the server. To reflect the special needs of older adults, we used large font sizes and big buttons based on the design principles [33] to design the user interface for the smartphone app. We built the app using Apache Cordova [31], an open-source framework on the Mac OS X 10.11 (Apple Inc) machine. To facilitate communication between smartphones and the server, we used open-source libraries provided by Apache Cordova [31]. Figure 1 illustrates the screenshots of the user interface on smartphones when study participants captured, reviewed, and transmitted images of food items.

### Development of a Viewer App for Dietitians

**Figure 2:**
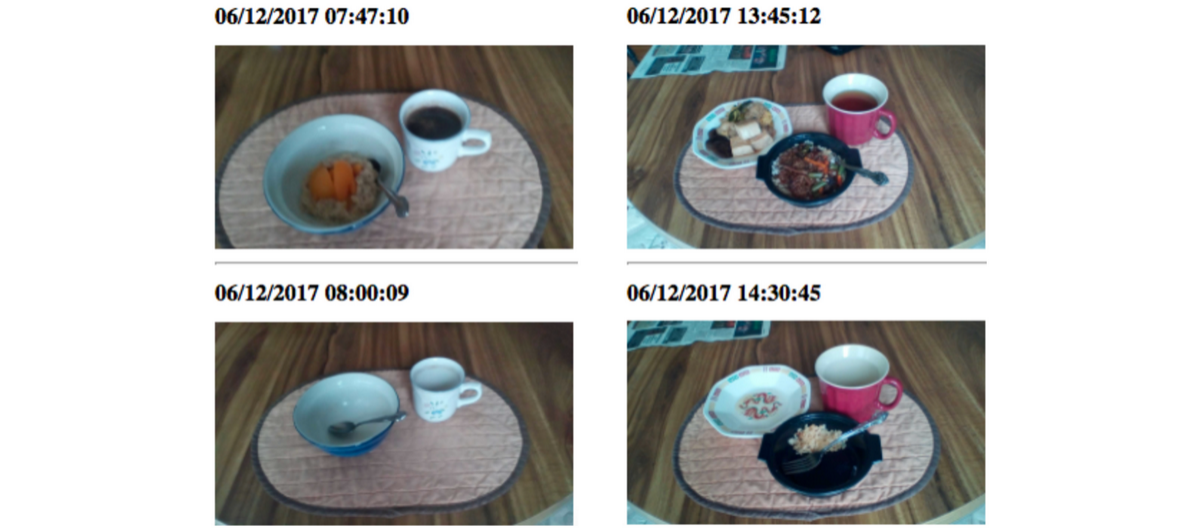
Screenshots of the user interface of the viewer app for reviewing images of food items during 24-hour dietary recall interviews.

A viewer app was designed to enable dietitians to view images of food items transmitted by smartphones of older adults with diabetes during 24HR interviews. We used HTML and JavaScript languages to develop the web-based app. The app was designed to perform two tasks: (1) gather images being stored in the database [30], and (2) display images to both dietitians and older adults with diabetes through devices (eg, laptops, tablets, or smartphones) during 24HR interviews. As Figure 2 illustrates, the app displays a series of images transmitted by each older adult participant with diabetes. The date and time information were added to the top of each image as metadata for the images. In particular, the app allowed users to navigate the images taken more recently by scrolling down the page, as images were organized in chronological order.

### Participants in Evaluation of Usability of FRADA

The inclusion criteria for participants were as follows: (1) 65 years or older; (2) diagnosed with type 2 diabetes or prediabetes for at least 6 months; (3) understand spoken English; (4) have their own smartphones with cameras; (5) live in independent living facilities, such as retirement communities, retirement homes, senior centers, or senior housing; and (6) have experience with smartphone usage for at least 6 months. We excluded any participants who were legally blind or had severe auditory impairments to avoid any potential sample biases that might influence the study. All procedures were approved by the Institutional Review Board at the University of Washington (IRB ID: STUDY00000432).

### Recruitment Process

We recruited 14 participants to discover usability issues of FRADA. Twelve participants were recruited from three senior centers, two senior housing locations, and one community center located in the metropolitan area of Seattle, Washington, while two were recruited by our personal network through a mailing list of the Greater Seattle Dietetic Association and in person. Although the sample size was small (N=14), it was comparable to similar published studies designed to discover usability issues using a qualitative approach [34-36].

### Study Procedure

**Figure 3:**
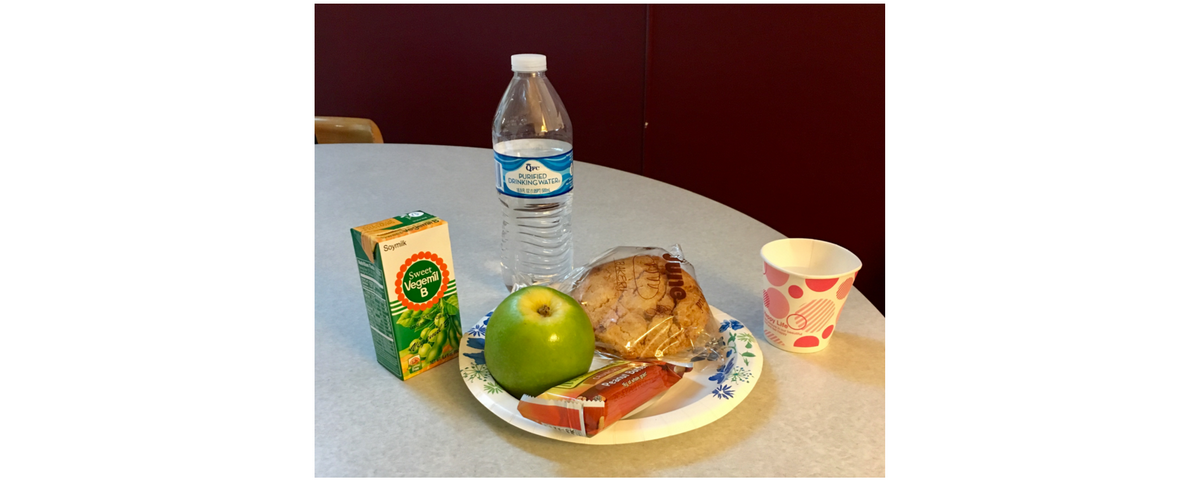
Food items used in the study.

We collected data from older adults with diabetes via a lab-based usability session that consisted of a pretest survey, a posttask survey, and a posttest interview. Before each test, we provided a smartphone (ie, iPhone 6; Apple Inc), a plate of food and beverages (see Figure 3), and instructions that included a list of rules that each participant should keep in mind when interacting with FRADA. The rules were as follows: (1) you should include all of the food items in one single photograph, and (2) to get the best images, you should hold the phone at a 45-degree angle when taking pictures of the food and beverages. After that, we asked each study participant to fill out the pretest questionnaire with demographic questions to get information about him or her, including age, gender, education level, and years of experience using a smartphone. We then proceeded with the lab-based usability session.

**Figure 4:**
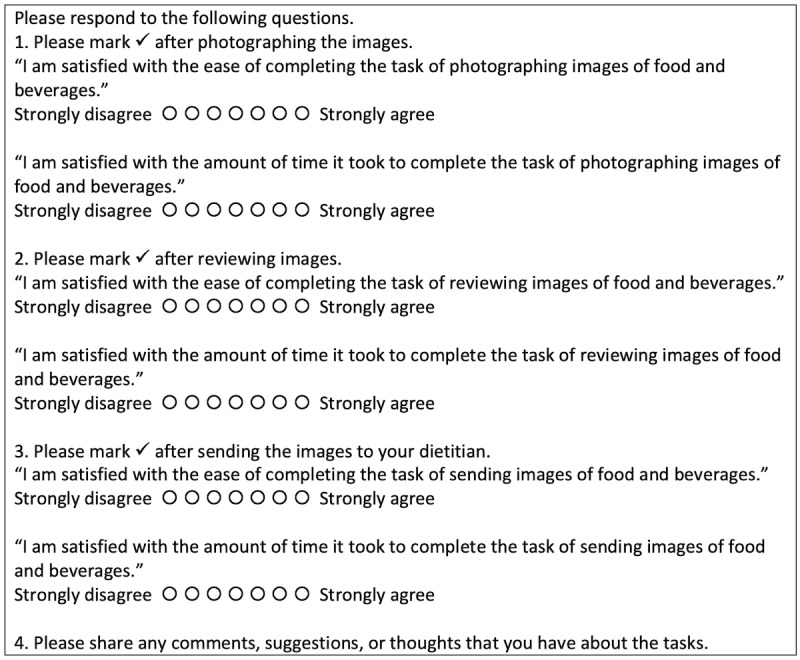
After-scenario questionnaire for older adults with diabetes.

During the test in the lab, each participant was asked to accomplish the tasks (ie, capturing, viewing, and transmitting images of food items) by interacting with FRADA (see Textbox 2). In order to answer RQ1 and RQ2, once participants completed the tasks, they were asked to fill out an after-scenario questionnaire (ASQ; see Figure 4), which is known to be a valid and reliable scale for usability tests [37] to rate the satisfaction of the ease of completing each task and the amount of time it took to complete each task using the smartphone app. The three statements of the ASQ, which uses a 7-point rating scale, are shown in Figure 4.

**Scenario proposed to participants during lab testing.**

You have an appointment with your dietitian tomorrow to assess your diet. The dietitian wants to get the smartphone images of the food and beverages that you have consumed before the appointment for your dietary assessment. Now, you need to accomplish the following three tasks using your smartphone:

- Capture an image of all the food and beverages served for your lunch.
- Review the captured image to make sure the image complies with the predefined instructions.
- Send the reviewed smartphone image of your lunch food and beverages to your dietitian.

After each participant completed the posttask questionnaire, we conducted a posttest semistructured interview with them in order to answer RQ3 through RQ10. The topics of the interview questions addressed the participant’s experience when he or she used the app to perform each task (see Textbox 3). The interview questions did not ask about relative preference for mobile app versus 24HR versus written food records, as the study participants did not compare these different tools directly. Each interview lasted 20-30 minutes and was conducted in person. All interviews were audiorecorded and transcribed.

**Sample posttest semistructured interview questions for older adults with diabetes.**

- What are your overall impressions of the smartphone app?
- If you had to give the smartphone app a grade, from A to F, where A was exemplary and F was failing, what grade would you give it and why?
- Name three words or characteristics that describe this smartphone app.
- What are the three things you like best about the smartphone app?
- What are the three things you like least about the smartphone app?
- If you could make one significant change to this smartphone app, what change would you make?
- Would you return to this smartphone app on your own in the future? Why/why not?
- What would entice you to return?
- Are there materials you would like to see added to the smartphone app? Which ones?
- Would you recommend this smartphone app to a colleague? To a friend?
- Do you have any other questions or comments about the smartphone app or your experiences with it?

### Data Analysis

To analyze the responses from the ASQ, we summarized the numbers and percentages of ratings and responses. Regarding the analysis of qualitative data from the interviews, we used an open coding method guided by grounded theory [38]. As a first step, the lead researcher of this study (HJ) read the transcribed interview text as a whole and highlighted statements that included important information (ie, participants’ experiences, preferences, and impressions). After reviewing the highlighted statements repeatedly, the researcher marked each statement with an appropriate label. The labeled statements under the same concept were then grouped into categories based on the common properties, each of which corresponded to a research question we aimed to answer (see Textbox 1). The whole process of open coding was carried out by a single coder.

## Results

### Study Participants

Participant demographics are illustrated in Table 2. In total, 14 participants (11 female, 3 male) took part in the study. The average age of participants was 73.6 (SD 5.0, range 65-80) years. The education level of the participants varied: bachelor’s degree (n=5), master’s degree (n=3), high school graduate (n=3), professional degree (n=2), and other (n=1). Participants used mobile devices with various operating systems: Android (n=10), iPhone operating system (n=3), and Amazon Fire (n=1). Of the 14 participants, 8 used smartphones for 2 or more years, while 5 used smartphones for less than 2 years (data was not reported for 1 participant).

**Table 2:** Participant demographics.

ID	Gender	Age (years)	Race/ethnicity	Education level	Number of years using a smartphone
P1	Female	76	White/Caucasian	Professional degree	2-3
P2	Female	80	White/Caucasian	Master’s degree	>3
P3	Male	79	White/Caucasian	Bachelor’s degree	<1
P4	Female	72	Asian/Pacific Islander	High school graduate	>3
P5	Female	67	White/Caucasian	Bachelor’s degree	Not reported
P6	Female	65	White/Caucasian	High school graduate	>3
P7	Male	74	White/Caucasian	Professional degree	1-2
P8	Female	70	Asian/Pacific Islander	Bachelor’s degree	2-3
P9	Female	76	White/Caucasian	Master’s degree	>3
P10	Female	67	White/Caucasian	Bachelor’s degree	>3
P11	Female	78	White/Caucasian	Bachelor’s degree	<1
P12	Female	70	White/Caucasian	Other	>3
P13	Male	79	White/Caucasian	High school graduate	1-2
P14	Female	77	White/Caucasian	Master’s degree	1-2

### Survey Results

Survey results based on ASQ revealed that participants tended to be satisfied with the ease of completing the tasks of photographing, reviewing, and sending the images of food and beverages. In addition, it seemed that they were satisfied with the amount of time it took to complete the task of photographing, reviewing, and sending the images of food and beverages. The average scores for completing the task of photographing, reviewing, and sending the images of food items were: 6.8 (SD 0.6), 6.9 (SD 0.3), and 6.9 (SD 0.3) out of 7 points, respectively. In particular, 12 out of 14 participants reported 7 points for all of the questions.

### Interview Results

Qualitative data were organized into eight categories through qualitative analysis steps guided by grounded theory [38]: (1) usability of FRADA, (2) potential benefits of using FRADA, (3) concerns of older adults, (4) willingness to use FRADA, (5) potential features to be added to FRADA, (6) easy instructions, (7) tablet as an alternative device, and (8) potential target populations.

#### Usability of FRADA

Participants reported that it was efficient to perform tasks such as capturing, viewing, and transmitting images of food items using FRADA. They also stated that it was easy to interact with the user interfaces of FRADA. P9 stated, “There’s only three moves basically, you know. Take a new picture, take the picture, and send the picture. It’s very efficient. I can’t think of any suggestions to make it better.” P4 noted the simplicity of the process of taking and sending a picture of food items: “Well, probably just take a picture and sending it to a dietitian in one step.” Furthermore, participants reported that FRADA was simple to use to perform the required tasks. P6 stated, “A+. It’s very simple and very easy to use.” P14 stated, “So, I think it’s very, very useful. I think it’s very worthwhile and excellent thing that you’ve come up with. And, as with all things that are excellent, it’s simple.” Moreover, P12 addressed the usability of the app by comparing it to other apps he had used: “Oh, yeah, well, like I said, it’s so much easier than anything that I’ve tried. I’m looking forward to seeing how you develop this app.” In particular, P1 and P11 reported that they did not have any difficulty in interacting with the app. P1 stated, “No, I didn’t have any difficulty.” P11 said, “It’s very easy to use. It’s very intuitive. I had no problem with it at all.”

In addition to the efficiency of using FRADA, participants mentioned that they were satisfied with the app’s user interface. For instance, the majority of the participants liked the large font size of the letters on the app. P2 said, “But, I did notice that your app has large letters, so it’s easy to read. You don’t have that little, teeny letter you have to look at.” P10 mentioned that the large print will help potential users of the app: “It was very easy to use. And the large print, I think is very helpful to people who have a hard time seeing. It was, it was easy. I, I—it was easy to do.” Similarly, P11 enjoyed using big buttons on the user interface: “The app buttons, the big buttons, they were very large, which for me, another guy can see that for an elderly person because in texting with a phone I don’t have small enough fingers or touchy enough fingers to do it well, but these buttons would make the app very easy to use for me.”

#### Potential Benefits of Using FRADA

We discovered that FRADA is perceived as beneficial in multiple ways. For instance, we learned that the images of food items collected by FRADA might bring about improved communications between older adults with diabetes and health care providers. In addition, we found that FRADA may be used to improve participants’ personal health and their experiences with keeping track of food items, as well as manipulating a smartphone.

First of all, participants stated that FRADA would likely facilitate improved interactions with health care providers. P11 stated, “Apparently this is going to send data to a dietitian and the dietitian then can give the person using it feedback on what they might do to correct their diet, too, I guess that’s what it is...so it’s an ongoing relationship between the patient and the dietitian.” P10 said that the images from the app might support the practice of dietitians: “...because sometimes we don’t tell the dietitian everything, but this way she can see everything we ate... It would be something that would be very helpful to people when, when discussing their, their eating habits with a dietitian.” Similarly, P6 mentioned that health providers would benefit from the images collected by the app: “...because it’ll help people deal with their dietitian and their doctor at the same time. So that everybody can get together and see what you’re doing.” P12 mentioned that dietitians will be able to provide service by using the collected images of food items: “...And then it goes to the dietician, and the dietitian’s gonna be able to give me information.” P1 also mentioned that the app would be beneficial to meeting with a dietitian: “But if I—as I go through the day, if I just clicked a picture each time, I think that would be valuable, so not only meeting with the dietitian, but just for my own.”

Further, participants stated that using FRADA would likely help them improve their personal health. P9 anticipated that FRADA would be valuable “in helping somebody to decide how to change their diet for optimal health.” Similarly, some participants expected improved health after using the app. P5 stated, “You know, if they asked me to take pictures, if they were asking me to use the app, I think I’d eat even better.” P14 said, “But this has a function that’s directly related to promoting my health. So, that’s a clear, pragmatic use.” Two participants mentioned that FRADA would improve their experiences with keeping track of meals and snacks. P9 stated, “This would be faster if I use that. Take a picture of what you normally eat or what you just ate for your lunch and tell me if that’s a good example of what you would have... In that case, I could do it and it would be very fast. Much faster than seeing a handwritten or a computer-generated list of what they had.” P1 described the process of taking pictures of food items by comparing with her existing practice of keeping a food diary: “Like I said, keeping a food diary is just so boring, but if I could take a picture several days in a row –– then I could just write all those things down, in my food diary.” In particular, P9 mentioned that the images captured by the app provide information describing the actual project: “Instead of serving size, then you see the product, the actual product.”

In addition to the direct benefits of using the app, P14 stated that the action of using FRADA enabled him to learn to take pictures using his smartphone—an indirect benefit: “Well, I like it because it gives me practice taking pictures with my smartphone because I generally use a camera and not a smartphone. But here, I’d be doing it frequently. So, I would have experience doing it. And I like that sort of side effect of the app that it’s teaching me.”

#### Concerns of Older Adults

We identified participants’ concerns about the use of FRADA. One of the critical concerns that participants expressed was the potential financial costs that might occur when they interact with FRADA for an image-assisted dietary assessment. Other identified issues included (1) whether the collected data would be shared with other people appropriately, (2) whether older adults would be able to use FRADA effectively in real-world scenarios, and (3) whether health care providers would look at the data shared by older adults in detail.

Participants expressed concerns about their potential use of FRADA. Most participants were concerned about the costs they might incur when scheduling a meeting with a dietitian and purchasing FRADA. P11 stated, “I mean that I can see that if that’s going to be a continuous thing it would have to be almost, like, a doctor’s relationship where depending upon how much time the dietitian spends on your particular diet.” P5 mentioned, “My only concern was how much would it cost you to have to have a nutritionist or doctor look at it, see.” P1 was aware of how expensive it would be to involve a dietitian, although she uses FRADA: “Well, I just wondered how you would get the dietitian involved. You know dietitians are expensive.” Also, P12 was concerned about the cost of the app itself: “That’s huge. That’s huge. We’re seniors. You know, we live on Social Security, so I don’t have a lot of money. Any of the apps that I have are free.”

In addition, participants mentioned trust issues regarding food images collected by FRADA. For instance, P7 did not believe that all of the images reflect the truth: “You don’t know what’s in there (cup) ... Vodka.” Similarly, P2 said, “You’re going to end up picking your own favorite foods anyway. I mean, just like you have your favorite foods.” In addition to the accuracy of the images of food items reported by patients, P7 was uncertain whether the system would affect adherence: “A lot of patients aren’t cooperative. Patients do what they want. When I prescribe drugs for a patient, what percentage of them do you think take the drugs like they’re supposed to? What percentage of patients follow the instructions, for example with pharmacy, what percentage would you guess?” Furthermore, participants were concerned about other issues, such as privacy, learning how to use FRADA, and dietitians’ concerns. Regarding privacy, P2 stated, “Yes. And, truthfully, to me that would be eventually, for some people it’s going to work, for me it’s invasion of my privacy...it’s just personal. That’s a personal thing.” Although P2 was concerned about privacy, she still wanted to use FRADA if needed: “I would probably share now and then. If I was really having a problem, I probably would be more willing to share.” P7 was particularly concerned about whether other older adults could use this app. P1 was worried about dietitians’ concerns: “The only thing I might wonder about is if the dietitian would like to be able to see the ingredients on anything instead of just looking at the title, especially if she wasn’t familiar with that particular item.”

#### Willingness to Use FRADA

We discovered that the request of health care providers might be essential to motivate older adults with diabetes to use FRADA continuously in the future. Also, we learned that older adults with diabetes might not want to keep using FRADA if they do not have a strong, external motivation to use it.

Participants expressed that they were willing to use FRADA potentially in the future if FRADA was required to manage their health. They said that they would like to use the app if asked by health care providers. P1 said, “If it [FRADA] was available and the dietitian was available, it would be a great learning device and diagnostic device, for what you were eating, to how you could improve your diet habits.” P5 expressed interest: “Sure, especially, you know, if I had a nutritionist or doctor who wanted me to send them pictures, I’d be happy to.” P4 stated, “I would use the app if dietitian asks me.” P3 stated that he would choose to use the app if there were known benefits of using it: “I—well, I would return it, if it was part of a health, ongoing health procedure that I wanted to be involved with to, you know, to see that I was eating the right things, that’s all, because I presume when this goes in you get feedback on it, saying this ice cream cone here may not be a good idea. I don’t know.”

Nevertheless, P2 stated that she would not want to use FRADA in the future, as she was still satisfied with the process of using a food diary: “I think it’s easy to use. I personally probably wouldn’t use it because I don’t mind writing things down.” Even though P2 did not want to use the app in the future, she was satisfied with its usability: “It’s a very easy. It’s a really easy app to use because once you understand it and, as I said, it probably would work for some people. Not necessarily for me, that’s all.”

#### Potential Features to be Added to FRADA

We found that participants were interested in a potential feature that would enable them to exchange health information with health care providers. For instance, they wanted to receive feedback on the collected images of food items from dietitians. In addition, they were eager to supplement any missing information from the collected images transmitted to health care providers.

Participants reported that they wanted to have a space to communicate with health care providers, such as receiving information from health care providers and supplying them with additional information. P3 and P12 wanted to view any feedback on the images of food items from health providers. P12 stated, “…having your app that you just have to snap the picture without having to do the labels, just take your picture. And I’m not sure how you would do that with the dietary feedback from the dietitian. I’m not sure how that would work, but it would make it easy.” In particular, P3 emphasized the importance of getting feedback from health care providers: “I mean if you don’t get any feedback the whole thing is worthless.”

While many participants wanted to get feedback from dietitians, P2 wanted to provide dietitians with additional information about the portion sizes of the food items. To supply accurate information of food items, P2 stated, “...I think you’ve got about the right size of plate, but I would definitely check that out... I don’t have that knowledge. I’m sure that being—this is kind of a national brand to it, so they probably have a pretty good idea what’s in that, too.” Similarly, P10 was eager to supply additional information by leaving comments on the images of food items: “And if there was maybe a place on the app where you could put in a comment—a comment would be nice, I only ate half of this meal.”

#### Easy Instructions

Participants reported that the instructions that were provided on how to take, review, and transmit the images of food items using the app met their needs. The instructions given for taking images of food items were to (1) include all the food items in one single photograph, and (2) hold the smartphone at a 45-degree angle when taking the images of food items. Participants felt that the instructions for taking, reviewing, and transmitting the images of food items using the app were easy to follow. P14 stated, “Probably A [grade] because it’s so clear. The instructions are clear. And the instructions on being able to accomplish the task is very clear. I feel like I could accomplish it with one request to do so.” P9 mentioned, “Oh, I think it’s great. It’s very easy to learn and the instructions are good.” In brief, study participants felt that the instructions for capturing images of food items using FRADA were simple and easy to follow.

#### Tablet as an Alternative Device

Participants reported that they were interested in interacting with a tablet as an alternative device for taking, reviewing, and transmitting images of food items.

Although most participants were satisfied with the user interface of their smartphone, three participants expressed interest in using a tablet for performing the tasks of an image-assisted dietary assessment method. P10 said, “I mean it’s bigger, you know, but it’s still easy. It’s still easy to use. It’s as easy to use as my phone is.” P2 stated, “But, the tablet’s bigger, but it still works. It would probably work the same.” P14 expressed her interest in using the app on both her smartphone and her tablet: “Well, the only change that I would like to make is I have both an Android phone and an Android tablet. And my tablet, I would hope that this app can be available on the tablet as well.”

#### Potential Target Populations

Our study revealed multiple groups of potential users who could benefit from using FRADA. In addition to older adults with diabetes, potential target populations include patients with diabetes, newly diagnosed patients with diabetes, and individuals sensitive to food intake. First, participants agreed with the statement that this app would support patients with diabetes. P1 stated, “I would recommend it to people with diabetes. I don’t know what other people would use it for, but people with diabetes, pre-diabetes.” Also, P2 expressed her recommendation: “I would recommend that diabetes patients use this app.” Next, participants mentioned that FRADA could be particularly beneficial to newly diagnosed patients with diabetes. P2 said, “I guess, it’s very useful, as I say. I think for a new diabetic, it would be very useful. A person that’s been newly diagnosed with diabetes.” In addition, participants reported that a potential target user population might be individuals who are sensitive to food intake in addition to patient with diabetes. P2 said, “Especially if they were, again, I’m talking about new diabetics or people who are trying to, or even people who are trying to lose weight and need to work with a dietitian.” P10 suggested that FRADA could benefit people after surgery “because they need to manage their diets and they need to talk with their nutritionists.”

## Discussion

### Principal Findings

Through surveys and interviews with older adults with diabetes in a lab-based usability session, this study revealed that participants tended to be satisfied with the usability of the newly developed FRADA and its instructions after performing three tasks (ie, taking, reviewing, and transmitting images of food items) successfully. Even though responses from participants revealed some concerns about interactions with FRADA, they indicated a willingness to use FRADA based on their needs in the future and identified additional target populations who could benefit from the use of FRADA (ie, patients with newly diagnosed diabetes, high blood pressure, or chronic kidney disease). Based on the findings of this study, there are topics that need to be considered, such as the appropriateness of existing or emerging modalities for older adults with diabetes, instructions, device preferences, target users, understanding multiple stakeholders’ needs for better tool design, and a cost-sensitive population.

#### Appropriateness of Existing or Emerging Modalities for Older Adults with Diabetes

While this study focused on recording images of food items as logs, there still exist a variety of modalities, such as wearable cameras, voice recordings, and sensor technologies that might be appropriate for older adults with diabetes to record their meals and snacks. First, wearable cameras may be appropriate for the older adult population. Since images are captured automatically by wearable cameras [22,28], older adults might not have to learn how to manipulate the devices. Similarly, older adults do not have to follow the instructions (ie, including all food items in one photograph, holding the phone at a 45-degree angle, and capturing images before and after eating events) when collecting images of their meals and snacks. Instead, older adults would only need to learn basic functions, such as how to turn the camera on and off and how to charge it. Even though wearable cameras may reduce the burden on older adults, users might still need to screen collected images prior to analyzing the images. In addition, low-income older adults might not want to purchase wearable cameras for the sole purpose of collecting images of food items.

Next, a voice-recording strategy might work well to support the older adult population. Similar to wearable devices, older adults do not have to follow the instructions that are required for collecting images of food items. Instead, they could simply turn on the voice recorder when logging food intakes. Although a voice-recording strategy may help older adults record food items and their portion sizes, there are still challenges that older adults might face when recording their voices. For instance, older adults would still need to turn on their voice recorder before eating events. Also, it might be difficult for older adults to describe every single food item and its portion size accurately. Similarly, sensor technologies (eg, a jaw motion sensor [39]) may be appropriate for older adults. For example, by monitoring chewing, a jaw motion sensor can detect periods of food intake automatically while people consume food [39]. This might be particularly beneficial to older adults because they do not have to perform any tasks to record food items and their portion sizes during eating events. However, it is still questionable whether such sensor technologies monitoring individuals’ eating behaviors might be socially acceptable for the older population.

#### Instructions

Participants were required to follow two instructions when taking pictures of food items. Although the instructions were based on the findings of previous studies, this study showed that the instructions would need further revisions to reflect the needs of health care providers. For example, dietitians might want to view the images taken at multiple angles for better understanding of the food items and their portion sizes.

#### Device Preferences

This study showed that some participants were interested in using the app on a tablet device, whose screen is larger than the screen of a smartphone. Since this study focused on evaluating the usability of the smartphone app, the findings do not indicate if older adults with diabetes actually prefer a larger screen. Nonetheless, it would be valuable to conduct additional experiments to determine the acceptability of other types of devices (eg, tablet, laptop, desktop, or smartwatch).

#### Target Users

We noticed that some participants did not want to use the smartphone app in the future because they were already familiar with how to manage their diabetes without it. Instead, they suggested that this app might benefit newly diagnosed patients with diabetes. This implies that researchers and designers might need to consider the unique needs of patients with other types of chronic diseases, such as high blood pressure and chronic kidney disease, when asking them to use technologies with mobile devices.

#### Understanding Multiple Stakeholders’ Needs for Better Tool Design

This study focused on evaluating the usability of FRADA with a single population of older adults with diabetes. As a result, we were able to identify potential features to be added to this app and the benefits of using FRADA based on the participants’ feedback. Future work would need to incorporate the needs of other stakeholders—such as dietitians, family members, friends, and caregivers—when selecting features to be added to this app and could reveal the benefits to these other stakeholders from using the app.

#### Cost-Sensitive Population

We found that most participants were sensitive to the potential costs involved in using FRADA. This might reflect the characteristics of participants in this study because they were mainly recruited at senior centers and senior apartments. There are more likely to be more low-income older adults in those facilities than in retirement communities with relatively higher living costs.

### Limitations

This study has a number of limitations that will need to be addressed in future work. One of the limitations is that there might be potential biases related to having only one coder in the qualitative data analysis. To analyze the qualitative data from the interviews, the lead author applied an open coding method [38] to collect statements from transcripts, identify recurring concepts, and group the statements into categories as a single coder. Thus, it is possible for a single coder to only accept categories from his or her own perspective and omit categories from other perspectives.

In addition, the study sample may not be representative of the larger target population (ie, older adults with diabetes). This is because our study only focused on older adults with diabetes who had prior experience with smartphones; some older adults with diabetes do not have previous experience with smartphones. Further, study participants were recruited only in the Pacific Northwest region of the United States. This sampling may limit how the study findings can be generalized.

Next, even though it was easy and efficient for older adults with diabetes to use FRADA for collecting images of food items, it is still questionable whether design implications from this study could be generalized and used for redesigning FRADA. For example, this study did not report the usability of FRADA with stakeholders other than older adults with diabetes. The findings of this study did not reveal needs and barriers related to other direct and indirect stakeholders (eg, dietitians, family members, friends, and caregivers) in the process of the image-assisted dietary assessment. Further usability testing with a more diverse sample in terms of race, ethnicity, literacy, health literacy, and technology experience would produce more generalizable insights.

Furthermore, special considerations for older adults with diabetes who also have cognitive or physical disabilities (eg, dementia or vision problems) were not addressed when we developed FRADA, although it is known that older adults with diabetes are twice as likely to develop dementia than older adults without diabetes [22]. Similarly, FRADA may not reflect the needs of older adults who have trouble holding a mobile device, as the user interface of FRADA was based on design guidelines [35] that address the needs of a general population of older adults rather than addressing the needs of people who experience specific functional or cognitive limitations (eg, limitations resulting from Parkinson disease).

While this study demonstrates the usability of FRADA for older adults with diabetes in a lab-based setting, there still remain questions about the validity of FRADA in a real-life setting. For instance, the accuracy of portion size estimation using FRADA is still unknown, as FRADA was not validated against an actual dietary assessment method (eg, 24HR interviews) at this time. Further validity testing involving dietitians could confirm whether using FRADA improves the accuracy of a traditional dietary assessment method, in which dietitians conduct 24HR interviews with older adults with diabetes.

### Conclusions

The goal of this study was to create a smartphone app that enables older adults with diabetes to collect images of food items and determine its usability. We achieved this goal via the development of FRADA and lab-based usability sessions with older adults with diabetes. Achieving this goal demonstrates three contributions. First, we created FRADA for an image-assisted dietary assessment based on design requirements that reflect the special considerations of older adults, which were not reflected in other apps used in previous studies. Therefore, designers, developers, and researchers could use the findings of this study to create smartphone apps targeting older adults with diabetes. Second, we obtained structured feedback about FRADA from participants through three types of questionnaires: pretest, posttask, and posttest using surveys and interviews. Our findings expand on existing knowledge about how to design smartphone apps for an image-assisted dietary assessment in older adults. Finally, we demonstrated the potential opportunities for evaluating the feasibility and validity of FRADA in a deployment study. While our study focused on evaluating the usability of FRADA by older adults with diabetes, further work remains to evaluate its usability with both direct and indirect stakeholders, such as dietitians, family members, friends, and caregivers to identify potential features to incorporate into FRADA. In addition, we plan to evaluate the feasibility and validity of an image-assisted dietary assessment using FRADA with older adults with diabetes so that we can determine if the method is clinically meaningful.

## Abbreviations

24HR: 24-hour dietary recall
ASQ: after-scenario questionnaire
FRADA: food record app for dietary assessments
MNT: medical nutrition therapy
